# Unwinding Complexities of Diabetic Alzheimer by Potent Novel Molecules

**DOI:** 10.1177/1533317520937542

**Published:** 2020-08-31

**Authors:** Sumeet Gupta, Anroop Nair, Vikas Jhawat, Nazia Mustaq, Abhishek Sharma, Meenakshi Dhanawat, Shah Alam Khan

**Affiliations:** 1 Department of Pharmacology, MM College of Pharmacy, MM (Deemed to be University), Mullana (Ambala), Haryana, India; 2 Department of Pharmaceutical Sciences, College of Clinical Pharmacy, King Faisal University, Al-Ahsa, Kingdom of Saudi Arabia; 3 Department of Pharmaceutical Sciences, G. D Goenka University, Gurugram, Haryana, India; 4 Department of Pharmaceutical Sciences, MM College of Pharmacy, MM (Deemed to be University), Mullana (Ambala), Haryana, India; 5 Department of Pharmacy, Oman Medical College, Muscat, Oman

**Keywords:** Alzheimer, diabetes mellitus, pharmacotherapy, new drug molecules, pathophysiology

## Abstract

Diabetes mellitus is one of the aggressive disorders in global society. No pharmacotherapy is available for permanent diabetes cure, although management is possible with drugs and physical activities. One of the recent complications noticed in type 2 diabetes mellitus includes diabetes-induced Alzheimer. It has been proposed that the possible diabetes-induced Alzheimer could be of type 3 diabetes. A variety of cross-sectional studies have proved that type 2 diabetes mellitus is one of the factors responsible for the pathophysiology of Alzheimer. New drug molecules developed by pharmaceutical companies with adequate neuroprotective effect have demonstrated their efficacy in treatment of Alzheimer in various preclinical diabetic studies. Patients of type 2 diabetes mellitus may show the benefit with existing drugs but may not cause complete cure. Extensive studies are being carried out to find new drug molecules that show their potential as antidiabetic drug and could treat type 2 diabetes–induced Alzheimer as well. This review provides an overview about the recent advancement in pharmacotherapy of diabetes-induced Alzheimer. The pathomechanistic links between diabetes and Alzheimer as well as neurochemical changes in diabetes-induced Alzheimer are also briefed.

## Introduction

**Figure 1.**
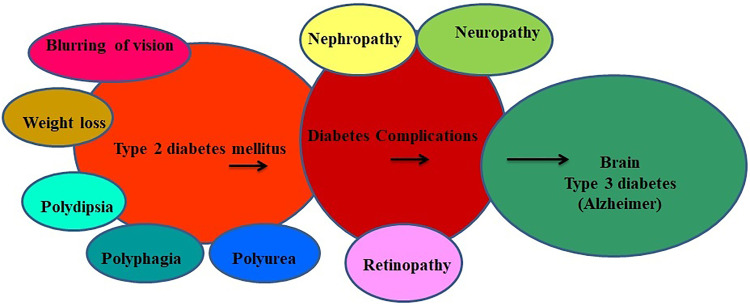
Schematic representation about the development of Alzheimer in type 2 diabetes mellitus.

Diabetes mellitus is one of the chronic disorders among noncommunicable diseases that affect a vast population in both developing and developed nations.^1^ Latest figures on diabetes is alarming, as it causes 4.2 million deaths globally while the health expenditure accounts to US$760 billion.^2^ According to World Health Organization, the global prevalence of diabetes in 2019 was around 463 million and predicted to rise up to 578 million (which represents the 10% of the worldwide adult population) by 2030.^2^ Diabetes mellitus consists of endocrine disorders characterized by hyperglycemia, changes in lipids, carbohydrates, protein metabolism, and gradually affects the central nervous system which can induce nervous system damage.^3^ Type 2 diabetes mellitus is connected with multiple risk factors (Figure 1), and about 60% to 70% of the diabetic individuals showed placid to rigorous forms of nervous system damage leading to Alzheimer disease.^4^ Ideal diagnostic biomarkers and efficient disease-modifying therapies for diabetes and its complications are still not up to the mark. Thus, development of tools which is easy-to-perform, low-cost diagnostic approaches, and disease-modifying therapies for diabetes mellitus has become the major concern for scientists in today’s medical world. In 2005, a scientist “Susanna” first suggested the name “type 3 diabetes mellitus” for diabetes-induced Alzheimer disease (brain) in patients with diabetes.^5^ The level of insulin increases in blood and incapable to show its action after binding on insulin receptor site.^6^ Hyperinsulinemia and insulin resistance are both the hallmarks of type 2 diabetes mellitus which can lead to memory impairment.^7^ Pathological conditions such as formation of neural-fibrillary tangles and β-amyloid deposition in the patient of diabetes mellitus brain clearly demonstrated and are responsible for the development of dementia and vascular type disorders affecting mainly in the central nervous system.^8,9^ The first study conducted on Japanese people reported that those who had higher blood sugar levels at an age of ≥60 years were 6 times more prone to develop protein deposit in the brain.^10^ From the past many years, researchers are showing more interest to explore the effect of chronic glucose level on neurological comorbidities in the brain. Symptoms of cognitive dysfunction and dementia are less popular and not well studied in the complications of diabetes mellitus.^11^ In some reported cases, memory impairment in the diabetic patients is higher when compared to individuals with normal blood glucose level.^11^ The present review summarized various pathomechanistic links between diabetes and Alzheimer with their future drug target molecules that may come into the market in the next couple of years.

## The Prevalence of Diabetes Mellitus and Associated Alzheimer

World Health Organization in 2019 reported that about 9.3% of the people had diabetes,^2^ and it would expand up to 11% by 2045.^2^ The prevalence of type 2 diabetes mellitus is much higher in developing countries than developed countries of which China and India share the largest contribution. Various retrospective population-based studies also provide an association between type 2 diabetes and Alzheimer due to hyperinsulinemia. Longitudinal studies found decline in cognitive action which increases from 1.5 to 2.0 times in individuals had type 2 diabetes mellitus. Similar results were observed by Rotterdam study, where they found about 126 patients having development of dementia of 6370 elderly diabetic patients, and 89 of these were specifically diagnosed with Alzheimer.^9^ Contrary to this result, another study reported no positive relationship between type 2 diabetes mellitus and accelerated cognitive decline in patients aged 85 years and older.^12,13^ A study on the Swedish population reported positive results for developing Alzheimer in men who developed diabetes mellitus at midlife age (32 years). These reports suggest that 100% chance to develop Alzheimer in diabetic individuals having a lack of insulin production than those with sufficient insulin production.^14^ Some studies also show significant association in diabetic patients who lack apolipoprotein E4 (ApoE4) and may lead to diabetes-associated Alzheimer.^15,16^ So maybe this is one of the markers for the progression risk factor for Alzheimer in patients with diabetes mellitus. A meta-analysis study reported by Beissel and colleagues indicates a relative risk increase from 1.4 to 2.4 for Alzheimer in the diabetic population conducted by 11 retrospective studies.^8^ Among 11 studies, 4 showed an increased risk of Alzheimer in patients with diabetes mellitus, and the remaining 7 studies reported nonsignificant results. The discrepancies in the results may be due to different techniques used with respect to interview based-medical history, review of the prescribed antidiabetic medications, oral glucose tolerance tests, random blood glucose levels, and measurement of glycosylated hemoglobin. But based on the reported investigations, it was concluded that diabetes may increase the risk of developing Alzheimer.^17,18^ Other studies also confirm the relative risk of Alzheimer in diabetic population when compared to nondiabetic. The middle-aged patients are more prone to develop Alzheimer than individuals with 65 years or more.^19-25^ Prediabetes is one of the significant symptoms to show the risk of diabetes and may lead to Alzheimer with increase in age. A small prospective study conducted on older patients having a mild cognitive impairment revealed that 15% of people with diabetes mellitus showed higher risk progression to dementia.^26^

## Pathophysiology of Alzheimer Disease Associated With Diabetes Mellitus

**Figure 2.**
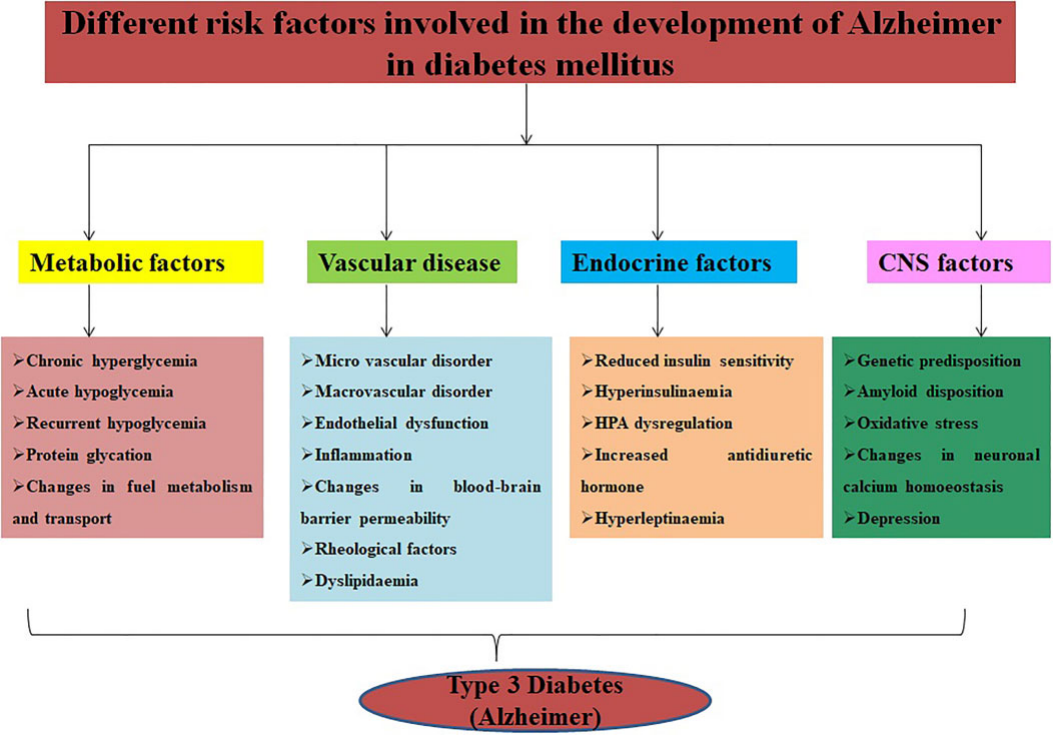
Various symptoms of type 2 diabetes mellitus and Alzheimer.

Different mechanisms are directly and indirectly involved in pathophysiology of diabetes-induced Alzheimer which includes insulin resistance, desensitization of insulin receptor, activation of protein kinase C and mitogen-activated protein kinase C,^27^ glucose metabolism, hyperglycemia, excess accumulation of advanced glycation end products (AGEs), mitochondrial dysfunction, amplified oxidative stress, receptor dependence diabetes, neurochemical changes (insulin-degrading enzyme, acetylcholine, amyloid and tau), inflammation markers, alteration in levels of growth factors, cytokines, and vascular.^28^ The major symptoms of diabetes mellitus include polydipsia, polyphagia, polyuria, blurring of vision, and weight loss due to insulin deficiency in the prediabetic stage (Figure 2). Recent studies reported 2 new physiological changes adding to diabetic complications are osteopathy and cognitive dysfunction. Leukoaraiosis is also one of the features shown at the age of 80 in patients with diabetes mellitus. The symptoms of leukoaraiosis demonstrated demyelination, increased water content, and gliosis.^29-31^ Several chronic symptoms were identified in patients having Alzheimer like the destruction of visuospatial skills and perception, worsening of mental ability, and relationship of language and progressive physical disability.^32^

## Role of Insulin in Alzheimer Disease

**Figure 3.**
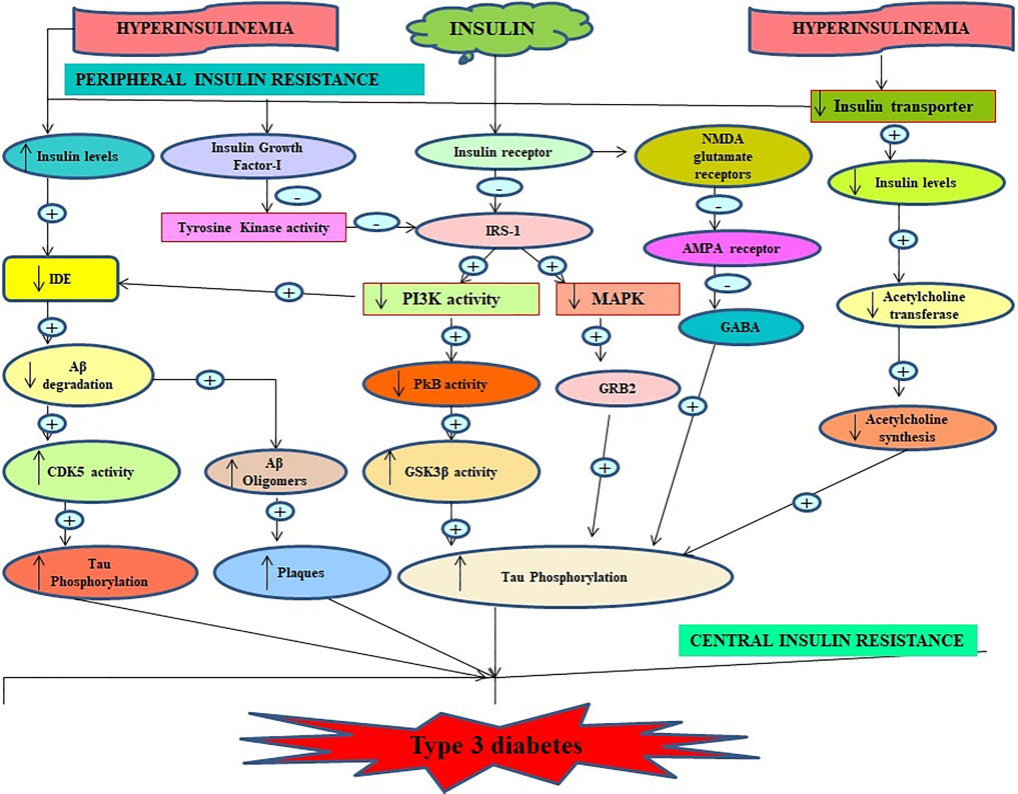
Important role of insulin in pathophysiology of type 3 diabetes (Alzheimer associated with diabetes mellitus).

Numerous evidences support that insulin can cross the blood–brain barrier by a saturable transport process mediated by the insulin receptor protein. The high concentration of insulin receptors available in the brain includes the olfactory bulb, hypothalamus, hippocampus, cerebral cortex, and cerebellum.^33,34^ Insulin and its receptors are the key factors in modulating glucose handiness in the central nervous system and peripheral systems (Figure 3). Alterations in brain glucose metabolism are one of the pathophysiological factors underlying this diabetic Alzheimer. Insulin-mediated phosphorylation of tau-protein stabilizes and accelerates the production of neurofibrillary tangles.^35-37^ Hoyer was the first scientist who suggested that condensed levels of brain insulin may disrupt cascade resulting in an alteration in cellular glucose metabolism, decrease acetylcholine and cholesterol synthesis, low production of ATP, impaired membrane function, depositing of amyloidogenic derivatives, and hyperphosphorylation of tau may lead to Alzheimer in patients with type 2 diabetes mellitus.^38^ Therefore, impaired insulin signaling may disrupt the physiological process of the amyloid β precursor protein, which may lead to type 3 diabetes, and these results support the hypothesis that amyloid β precursor protein contributes to Alzheimer neurodegeneration by impairing insulin signaling and insulin resistance.

## Role of Glucose and Thiamine in Type 3 Diabetes

**Figure 4.**
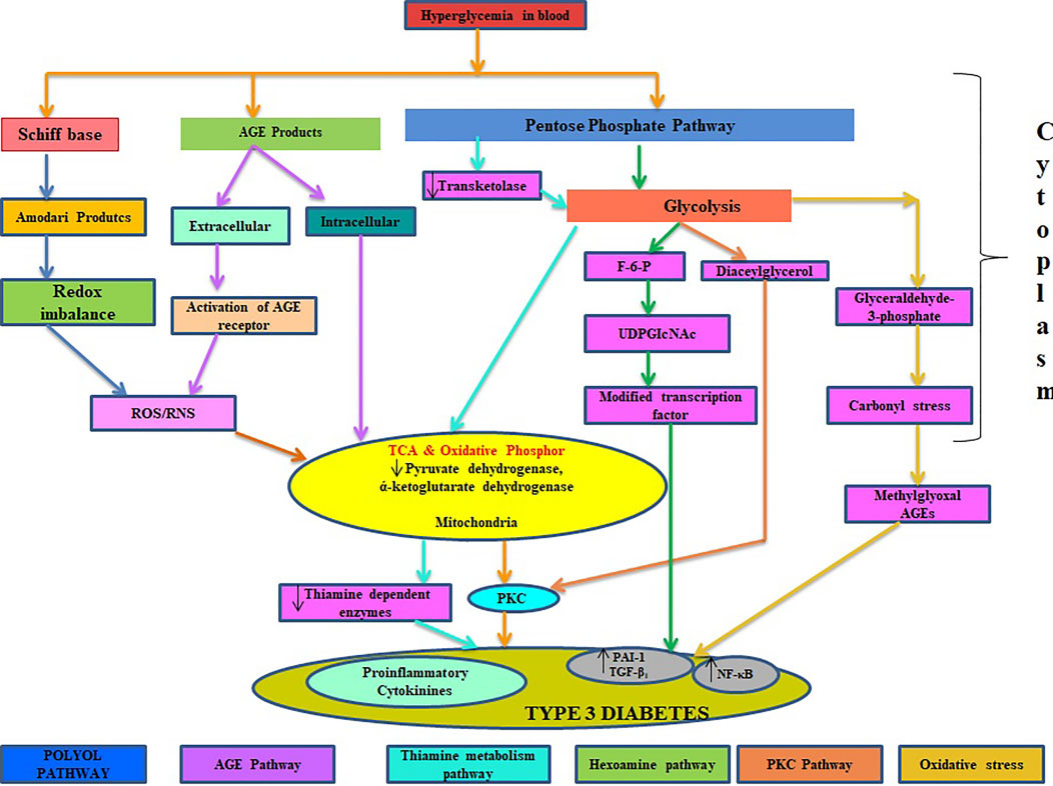
Physiological role of glucose in development of type 2 diabetes mellitus–induced Alzheimer.

**Figure 5.**
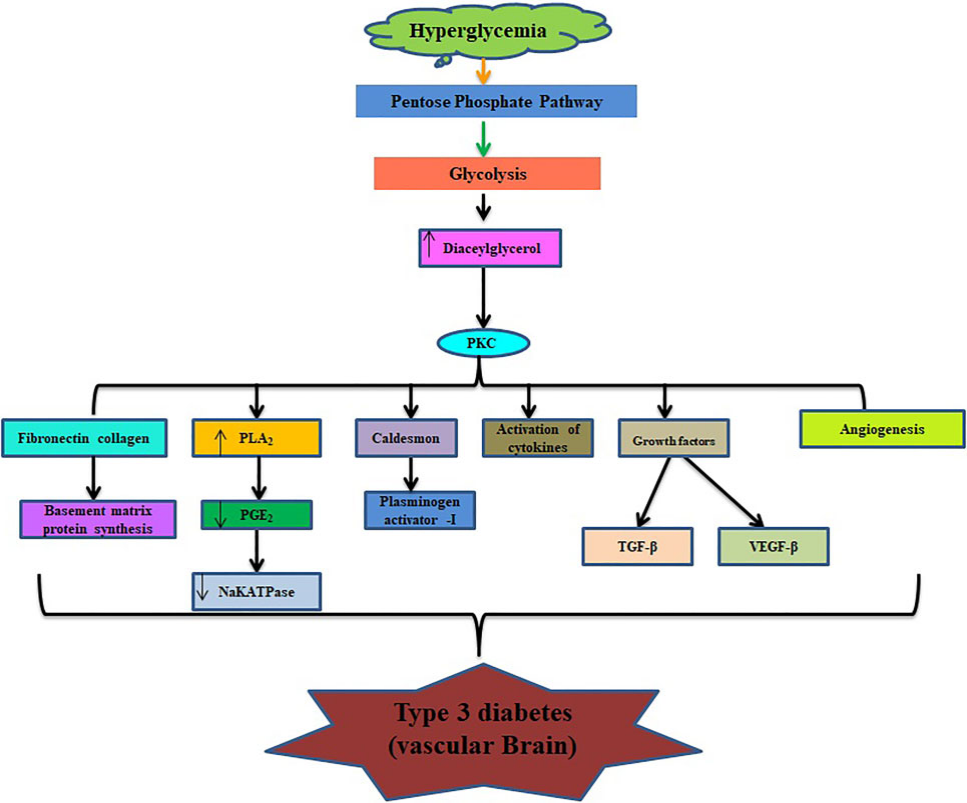
Role of glucose in vascular part of brain.

Glucose is the chief energy source for the human brain, but increased level of glucose (hyperglycemia; Figure 4) can lead to the development of diabetes and its complications such as Alzheimer. Cerebral glucose metabolism includes 2 main processes: glucose transportation and intracellular oxidative catabolism.^39,40^ Astrocytes play a significant role in adjusting glucose transportation and maintaining brain energy, a physiological state that promptly absorbs glucose from blood through the endothelial cells and conveys energy metabolic substrates between blood and neurons.^41,42^ GLUT-1 and GLUT-3 are thought to play essential roles in modulation of brain glucose transportation^40,43^ and development of Alzheimer.^43^ Disturbance of glucose transportation, mitochondria dysfunction, and vascular complications are well documented to cause Alzheimer (Figure 5). Glucose metabolism may provide some necessary compounds that include neurotransmitters such as acetylcholine and glutamate. Hypoglycemia is a common and severe side effect in the treatment of diabetes mellitus. The prolonged period at hypoglycemic stage may result in cognitive dysfunction in the form of neuroglycogenic, including difficulty in mental concentration, drowsiness, and in-coordination and may cause subclinical cerebral injury or permanent cognitive impairment.^44,45^ Thiamine also takes part in the regulation of oxidative and carbonyl stresses, and decreased activities of the thiamine-dependent enzyme are also one of the alternative clues to explore in the pathogenesis of mitochondrial dysfunction and abnormality in the metabolism of cerebral glucose.^46^ All of these mechanisms have a direct contribution to the microvascular changes in brain aging, which in turn results in cognitive decline and dementia.^47^

## Role of Inflammatory Cytokines in Type 3 Diabetes

Involvement of inflammation is well recognized in the pathogenesis of both diabetes mellitus and Alzheimer. First inflammation studies in patients having diabetes mellitus were seen 100 years ago when Ebstein observed that an anti-inflammatory drug sodium salicylate reduced glucosuria in patients with diabetes. Increased levels of inflammation markers such as fibrinogen, C-reactive protein (CRP), interleukin-6 (IL-6), plasminogen activator inhibitor-1 (PAI-1), sialic acid, and white cell count are associated with the type 2 diabetes mellitus.^48^ The Aβ peptide plays a central role in the neuroinflammation hypothesis of Alzheimer, which states that Aβ accumulation results in increased levels of inflammatory molecules (eg, cytokines, chemokines, and complement proteins) produced by chronically activated Glia. These results had implicated for the investigative tool of type 2 diabetes mellitus and Alzheimer.^49^

## Neurochemical Changes in Type 3 Diabetes

A significant linkage between diabetes mellitus, insulin resistance, and alteration in acetylcholine, apolipoproteins, amylin, and insulin-degrading enzyme were established in various studies. Ach synthesis is induced by the enzyme acetylcholine transferase. Low expression of acetylcholine transferase due to suboptimal levels of insulin and desensitization of insulin receptor may reduce the Ach in neurons of diabetic patients resulting in Alzheimer.^16,50^ Apolipoproteins are key enzymes in lipid metabolism, which deposit the neurotoxin Aβ, and certain isoforms of these apolipoproteins are responsible for the development of Alzheimer.^51^ Amylin is a first isolated enzyme obtained from amyloid deposits in patients with type 2 diabetes mellitus. This secreted protein travels with insulin through the bloodstream and gets deposited in the brains of the people having type 2 diabetes mellitus which was similar to patients with Alzheimer. Insulin-degrading enzyme insulinase was obtained from the liver extract and found to be responsible for the development of Alzheimer in insulin resistance/diabetic individuals.^52,53^

## Future Direction: New drug Molecules for Treating Diabetes and Alzheimer

### Glycogen Synthase Kinase-3 Inhibitors

Glycogen synthase kinase-3 (GSK-3) is a serine/threonine kinase enzyme that acts in glycogen synthase phosphorylation. GSK-3 regulates functions of various metabolic pathways through structural proteins and is capable of regulating many transcription factors including activator protein-1, cyclic AMP response element-binding protein, nuclear factor of activated T cells, heat shock factor-1, β-catenin, and nuclear factor κB (NF-κB). Positive link associated between GSK-3 and components of plaque amyloid protein system leads to Alzheimer. GSK-3 also participates in formation of microtubules binding protein tau that leads to the formation of neurofibrillary tangles that contributes Alzheimer disease. Additionally, GSK-3 regulates the neuronal plasticity, gene expression, and cell survival, which are key components of neurodegenerative diseases. Indeed, GSK-3 inhibitors are promising therapy in treating various neurodegenerative diseases.^54^

#### A-1070722

**Figure 6.**
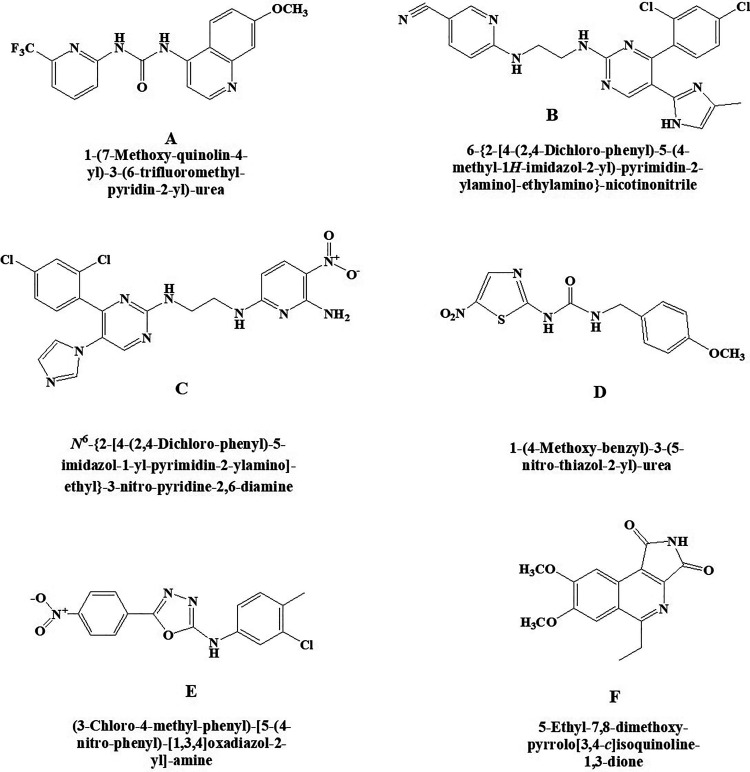
New glycogen synthase kinase-3 (GSK-3) inhibitors (A) A-1070722, (B) CHIR-99021, (C) CHIR-98014, (D) AR-A 014418, (E) TC-G 24, and (F) 3F8.

A-1070722 (Figure 6A) is a potentially effective compound that act as an inhibitor of glycogen GSK-3 which is helpful for the management of psychiatric and neurodegenerative disorders as reported by Baker et al.^55^ Chemically, it is 1-(7-methoxyquinolin-4-yl)-3-(6-(trifluoromethyl)pyridin-2-yl) urea. In vitro studies established that this molecule protects the primary cortical neurons of rat against β amyloid and glutamate challenge by declining phosphorylation of microtubule-associated protein tau.

#### CHIR-99021

A strong and highly selective inhibitor for glycogen GSK-3 exhibits >500-fold selectivity against insulin resistance (Figure 6B). Chemically, it is 6-((2-((4-(2,4-dichlorophenyl)-5-(4-methyl-1H-imidazol-2-yl)pyrimidin-2-yl)amino)ethyl)amino)nicotinonitrile. This GSK-3 inhibitor improved insulin-stimulated glucose transport in striated skeletal muscle of insulin-resistant Zucker diabetic fatty rats than insulin-sensitive lean Zucker rats. Findings suggested that this selective GSK-3 inhibitor may be useful for the treatment of the insulin resistance in type 2 diabetes.**^56^**

#### CHIR-98014

It is a selective and highly potent inhibitor of the GSK-3 (Figure 6C). Chemically, the compound CHIR-98014 is N2-(2-((4-(2,4-dichlorophenyl)-5-(1H-imidazol-1-yl)pyrimidin-2-yl)amino)ethyl)-5-nitropyridine-2,6-diamine. It is shown to inhibit the GSK-3β more specifically and improve insulin-stimulated glucose transport in both in vitro and in vivo. It is also shown to prevent the stimulation of TREM2 pathway, which is an important pathological factor in Alzheimer disease. This clearly indicates toward its potential use in diabetes.^56,57^

#### AR-A 014418

It is a kinase regulator of glycogen synthase kinase-3beta (GSK3beta) having numerous cellular functions (Figure 6D). Chemically, it is 1-(4-methoxybenzyl)-3-(5-nitrothiazol-2-yl)urea. It modulates microtubule dynamics affecting higher functions including cognition and mood and was investigated by Bhat et al.^58^ Therefore, the compound may have important applications to elucidate the role of GSK-3 in cellular signaling, especially in Alzheimer, as it does not extensively inhibit some closely related kinases like cdk2 or cdk5.

#### TC-G 24

It is an excellent potent inhibitor of GSK-3β having liver glycogen storage capacity in rodents (Figure 6E). Chemically, it is N-(3-chloro-4-methylphenyl)-5-(4-nitrophenyl)-1,3,4-oxadiazol-2-amine. In vitro and in vivo activities of this novel GSK-3β inhibitors was demonstrated by Khanfar et al.^59^ Dysfunctioning of GSK-3β is involved in the pathophysiology of many diseases such as type 2 diabetes mellitus, stroke, Alzheimer, and other related diseases.

#### 3F8

It is a selective GSK-3 type of inhibitor (Figure 6F). Chemically, it is 5-ethyl-7,8-dimethoxy-1H-pyrrolo[3,4-c]isoquinoline-1,3(2 H)-dione. It has shown more potential than SB216763 when assessed by in vitro kinase experiments (cell reporter assays) as reported by Zhong et al.^60^ Based on the structure of 3F8, a new therapeutic agent has been developed by synthesizing and validating by biological assays that can inhibit GSK-3. Hence, 3F8 and its derivatives could be potential new therapeutic targets for GSK-3–related diseases.

### Retinol-Binding Protein 4 Activator

Retinol-binding protein 4 (RBP4) are also known as retinol-binding protein stored in liver and adipose tissues. It acts as plasma retinol transporter (ROH) that carries retinol from liver to the periphery of the tissues via circulatory system. RBP4 has 2 isoforms in serum, namely, holo-RBP4 (RBP4 bound to ROH) and apo-RBP4, which remains after the release of ROH into the target cell. Imbalance between apo-RBP4 and RBP4 leads to pathophysiology of various diseases. Retinol-binding protein 4 plays a role in progression of insulin resistance through inflammatory markers. Increase in concentration of RBP4 induces neurodegenerative diseases. Retinol-binding protein 4 activator decrease the concentration of RBP4 and thereby improves insulin sensitivity in diabetes.^61^

#### A-1120

**Figure 7.**
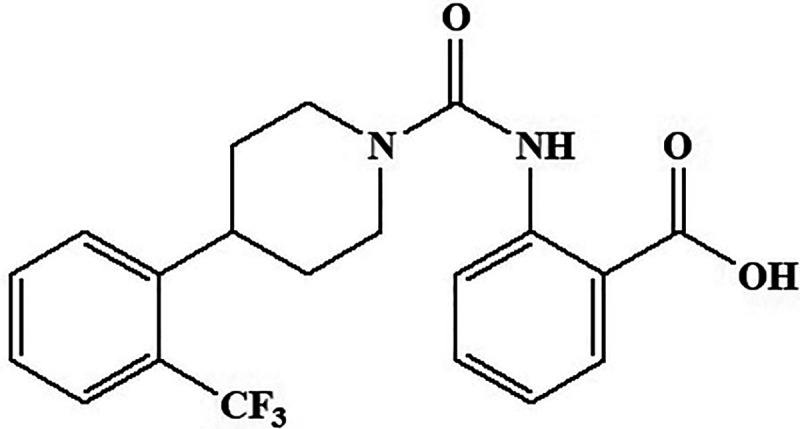
Retinol-binding protein activator compound A-1120.

A new molecule has been reported by Motani et al^62^ which acts on RBP4 with high affinity (Figure 7). Chemically, it is 2-(4-(2-(trifluoromethyl)phenyl)piperidine-1-carboxamido)benzoic acid. The occurrence of systemic insulin resistance is directly linked to an elevation in serum RBP4 that is secreted from adipose tissue. The molecule is said to improve insulin sensitivity by decreasing the retinol–RBP4 ratio. Therefore, the molecule can be a suggestive lead in diabetes-associated Alzheimer.

### AMP-Activated Protein Kinase

AMP-Activated Protein Kinase (AMPK activator; a master regulator of metabolism) is a heterotrimeric serine/threonine kinase enzyme and a key regulator of fatty acids as well as glucose homeostasis. Activation of AMPK results in phosphorylation and inactivation of acetyl-CoA carboxylase leads to prevent the formation of malonyl CoA. This enzyme helps long-chain fatty acid to travel into the mitochondria for oxidation. Inhibition of this enzyme via AMPK pathway is the main target for the treatment of diabetes and neurodegenerative diseases.^63^

#### A-769662

**Figure 8.**
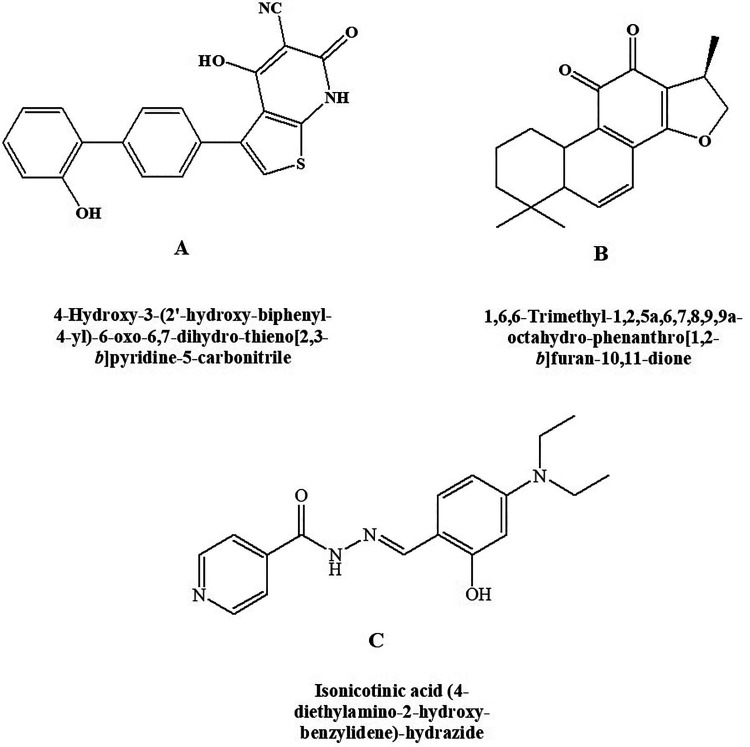
AMP-Activated Protein Kinase (AMPK activator) (A) A-769662, (B) cryptotanshinone, and (C) RSVA-405.

Type 2 diabetes mellitus and its associated disorders can be treated with potent and reversible AMPK activator. A-769662 (Figure 8A) is chemically 4-hydroxy-3-(2’-hydroxy-[1,1’-biphenyl]-4-yl)-6-oxo-6,7-dihydrothieno[2,3-b]pyridine-5-carbonitrile and is a potent AMPK activator having an EC_50_ of 0.8 μM as reported by Cool et al.^63^ In vivo studies depicted that glucose and triglycerides levels were reduced and inhibition of fatty acid synthesis by selectivity toward β1 subunit-containing heterotrimers.

#### Cryptotanshinone

Chemically, the compound cryptotanshinone is (R)-1,6,6-trimethyl-1,2,6,7,8,9-hexahydrophenanthro[1,2-b]furan-10,11-dione (Figure 8B). It is an AMPK agonist along with several other actions. Cryptotanshinone is shown to act as an antidiabetic agent producing its effect by activating AMPK. It is also found to be effective in obesity.^64^ In addition, cryptotanshinone was reported to reduce the β-amyloid deposition in vitro as well as in vivo through modulating α-secretase^65,66^ therefore indicating its efficacy in type 3 diabetes.

#### RSVA-405

Chemically, RSVA-405 is 2-[[4-(Diethylamino)-2-hydroxyphenyl] methylene] hydrazide-4-pyridinecarboxylic acid (Figure 8C). It is shown to possess AMPK activator property by acting on CaMKKβ-dependent mechanism in an indirect manner. The compound is reported to enhance the degradation of Amyloid β,^67^ inhibition of adipogenesis, and have high possibilities that it can produce an antidiabetic action through activating AMPK.^68^ Thus, it can be a beneficial agent in the management of type 3 diabetes mellitus.

## Farnesoid X Receptor

Farnesoid X receptor (FXR) is known as nuclear receptor super family (NR1H4) which is available in adrenals, intestine, kidney, and liver. It is also famous as nuclear bile acid receptor. Stimulation of FXR through chenodeoxycholic acid present in liver is most effective metabolism and controlling of bile acids. Targeting through FXR is the novel treatment for inhibition of insulin resistance and neurodegenerative diseases.^69^

### GW-4064

**Figure 9.**
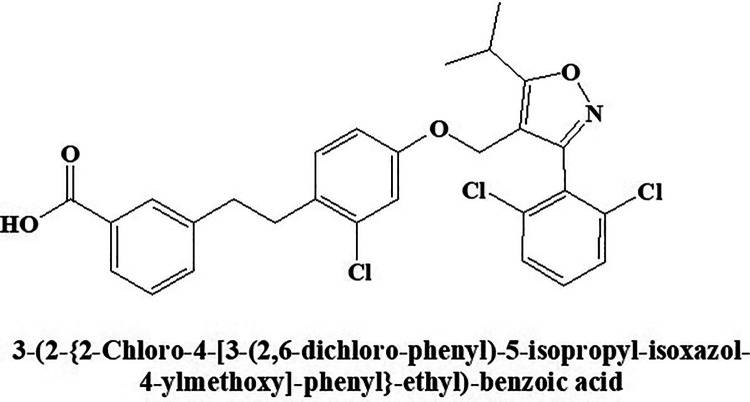
Nuclear receptor activator GW-4064.

Nuclear receptor FXR activator GW-4064 (Figure 9) improves hyperglycemia and hyperlipidemia in diabetic mice investigated.^70^ Chemically, it is (E)-3-(2-chloro-4-((3-(2,6-dichlorophenyl)-5-isopropylisoxazol-4-yl)methoxy)styryl)benzoic acid. Peripheral insulin sensitivity is improved by the molecule through FXR, while modulating adiposity in mice by the same molecule is reported by another researcher.^71^ This molecule also improves insulin sensitivity through insulin signals and mechanism of glucose uptake in treating differentiated 3T3-L1 adipocytes. This unforeseen utility of FXR opens a new viewpoint for the treatment of diabetes.

### Insulin-Mimetic Compounds

Increased glucose metabolism via tyrosine phosphorylation of the insulin receptor β subunit and phosphorylation of Akt resulting control the glucose levels in blood. Insulin mimetic type of drug suppresses the glucose levels via Akt pathway.^72^

#### Demethylasterriquinone B1

**Figure 10.**
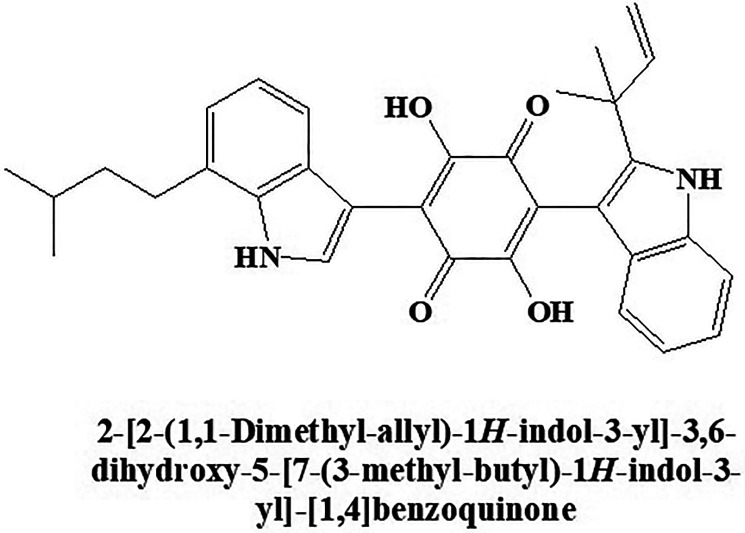
Insulin-mimetic compound Demethylasterriquinone B1.

The compound Demethylasterriquinone B1 (Figure 10) was isolated from an endophytic fungus known as *Pseudomassaria species.* showed insulin-mimetic action as reported by Webster et al.^73^ Chemically, it is 2,5-dihydroxy-3-(7-(3-methylbut-2-en-1-yl)-1H-indol-3-yl)-6-(2-(2-methylbut-3-en-2-yl)-1H-indol-3-yl)cyclohexa-2,5-diene-1,4-dione. It showed various insulin-like effects and inhibited glucose uptake in adipocytes and skeletal muscle tissue through insulin signal transduction and activation of Akt kinase.^73^ Research also shows that the insulin mimic action of Demethylasterriquinone B1 is due to the action on cellular target glyceraldehyde 3-phosphate dehydrogenase.

## Glycogen Phosphorylase Inhibitor

During contraction, glycogen phosphorylase enzyme catalyzes breakdown of glycogen to glucose-1-phosphate (2 forms) in liver and tissues. The less active B form is easily transferring to more active A form by enzyme phosphorylase. Drugs that inhibit this enzyme to prevent the convertible from glycogen to glucose-1-phosphate is the potential therapy for attenuating hyperglycemia associated with type 3 diabetes mellitus.^74^

### GPi 688

**Figure 11.**
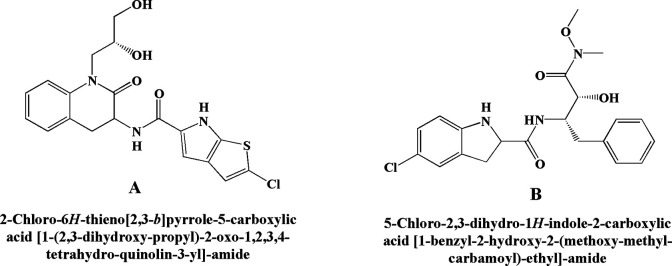
Glycogen phosphorylase inhibitor (A) GPi 688 and (B) CP 316819.

Chemically GPi 688 is 2-chloro-N-(1-((R)-2,3-dihydroxypropyl)-2-oxo-1,2,3,4-tetrahydroquinolin-3-yl)-6H-thieno[2,3-b]pyrrole-5-carboxamide. The compound acts on the indole site of glycogen phosphorylase, which is an allosteric glycogen phosphorylase inhibitor. It has been reported to inhibit glucagon-mediated hyperglycemia in vivo.^75^ Another researcher reported the inhibition of hyperglycemia activity mediated by increased glucose production.^76^ Findings suggested that in the clinical aspect, glycogen phosphorylase inhibitors (Figure 11A) may be more successful against fasting rather than a prandial condition.

### CP-316819

CP-316819 chemically is 5-chloro-N-((2S,3R)-3-hydroxy-4-(methoxy(methyl)amino)-4-oxo-1-phenylbutan-2-yl)-1H-indole-2-carboxamide. The study demonstrated the inhibitory activity of hepatic glycogen phosphorylase by this molecule for the treatment of hyperglycemia^74^ and transiently reduced muscle lactate production during a contraction but without affecting muscle energy metabolism. A study^77^ reported hypoglycemic activity in rats treated with the abovementioned molecule (Figure 11B) and showed an increase in brain glycogen content and acted as a novel glycogen phosphorylase inhibitor. From these data, it can be assumed that this molecule may give additional advantage for averting hypoglycemic coma and brain injury in patients with diabetes.

## c-Jun N-Terminal Kinase Inhibitor

Physiological stresses induced at the cellular level can leads to cell death via c-JUN N-terminal kinase (JNK) pathway. The stress-activated protein kinase members like JNK and p38 are involved in the phosphorylation and stimulates proto-oncogene *c-jun.* c-JUN N-terminal kinase has been linked to the regulation of proliferation, cell response, and cell death. JNK-3 is predominant in neurons leads to neurodegenerative diseases.^78^

### BI 78D3

**Figure 12.**
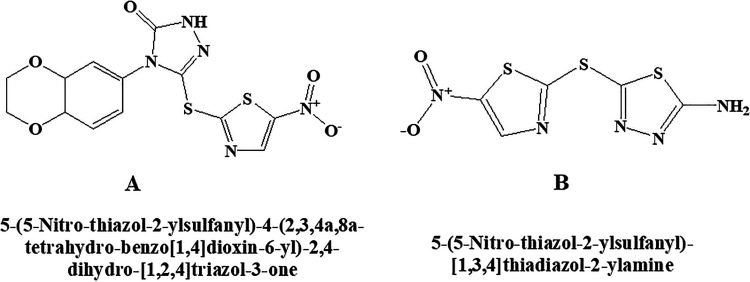
c-Jun N-terminal kinase (JNK) inhibitor (A) BI 78D3 (B) SU 3327.

BI 78D3 chemically is 4-(2,3-dihydrobenzo[b][1,4]dioxin-6-yl)-3-((5-nitrothiazol-2-yl)thio)-1H-1,2,4-triazol-5(4 H)-one. This molecule (Figure 12A) belongs to the category of competitive JNK inhibitor, which activates p38α preferentially rather than mTOR and PI-3K as reported in some of the studies.^79,80^ It also inhibits JNK interacting protein 1 (JIP1) and prevents JNK substrate phosphorylation. Blockage of JNK-dependent Con A-induced liver damage and restoring of insulin sensitivity in a mouse model of type 2 diabetes were reported.

### SU 3327

Chemically, the SU 3327 is 5-((5-nitrothiazol-2-yl)thio)-1,3,4-thiadiazol-2-amine (Figure 12B). Protein–protein interaction between JNK and JIP may restore insulin sensitivity. The effectiveness of the compound was reported in a mouse model of type 2 diabetes in the study^81^ producing its action by inhibiting JNK.

### Peroxisome Proliferator–Activated Receptor Activator

Peroxisome proliferator-activated receptor (PPAR-β) belongs to super family of nuclear hormone receptor and isomers of PPAR-α and PPAR-γ. Exclusively, PPAR-β is present in the brain, and disruption of this receptor leads to neurodegenerative diseases. Maintaining lipid levels in brain through activation of PPAR-β receptors results in neuroprotectivity.^82^

### GW-0742

**Figure 13.**
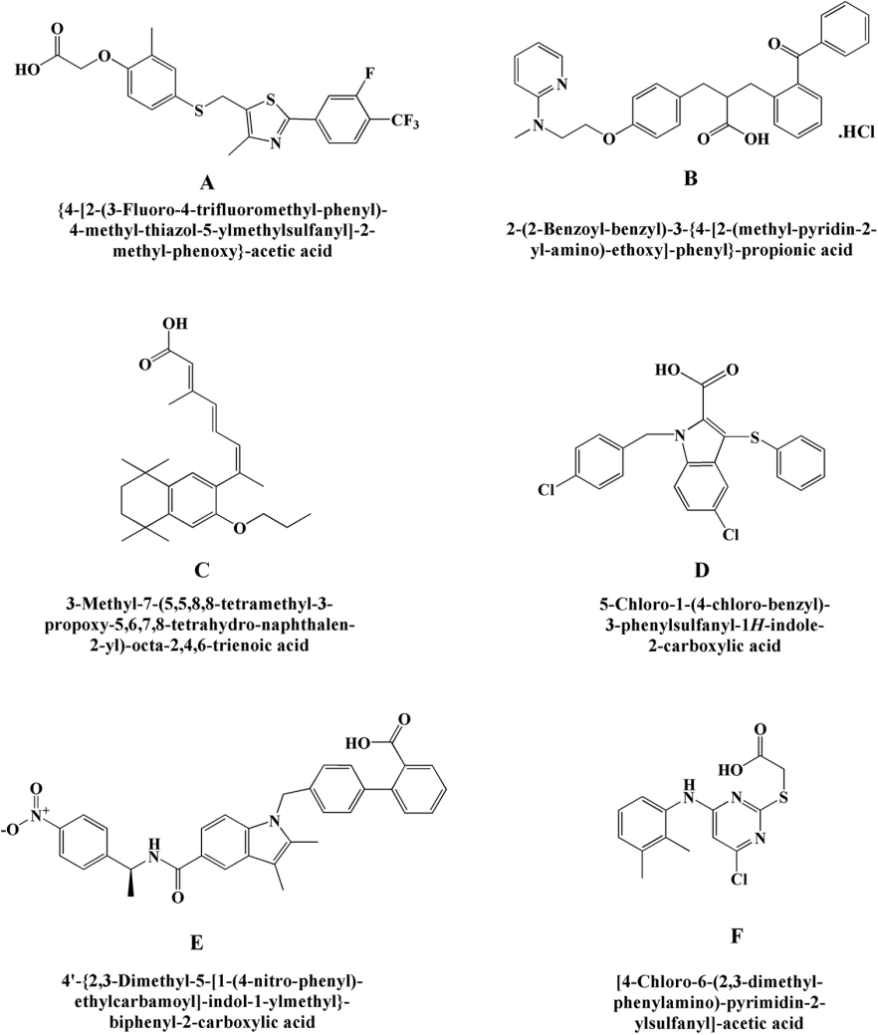
Peroxisome proliferator–activated receptor compound (A) GW-0742, (B) GW-1929 hydrochloride, (C) LG-100754, (D) nTZDpA, (E) SR-1664 and (F) WY-14643.

This is chemically 2-(4-(((2-(3-fluoro-4-(trifluoromethyl)phenyl)-4-methylthiazol-5-yl)methyl)thio)-2-methylphenoxy) acetic acid. It is a novel molecule that actively participates in the brain and can play a significant role in the regulation of genes in neurotoxicity via PPAR-β families. Sznaidman et al^83^ reported the protective activity of this novel molecule against diabetes and neuronal cell death through PPAR receptor families (Figure 13A). It was clear from the study that PPAR-β is worthy of further investigation to give a therapeutic target in neurodegenerative states.

### GW-1929 Hydrochloride

Chemically, GW-1929 hydrochloride is (S)-2-((2-benzoylphenyl)amino)-3-(4-(2-(methyl(pyridin-2-yl)amino)ethoxy)phenyl)propanoic acid hydrochloride (Figure 13B). The in vivo studies demonstrated that this molecule is PPARγ agonist showing a reduction in glucose, fatty acid, and triglyceride levels after oral administration. Long-term oral administration of this novel molecule to Zucker diabetic fatty rats showed dose-dependent decreases in daily glucose, free fatty acid, triglycerides, and glycosylated hemoglobin when compared to pretreatment values in in vivo studies.^84^ Hence, both in vivo and in vitro studies indicated that GW-1929 can be an effective agent.

### LG-100754

Chemically, LG-100754 is (2E,4E,6Z)-3-methyl-7-(5,5,8,8-tetramethyl-3-propoxy-5,6,7,8-tetrahydronaphthalen-2-yl)octa-2,4,6-trienoic acid. It is a novel compound that acts as a PPARγ agonist by enhancing its ligand-binding activity to decrease glucose levels. It also activates RXR PPARα heterodimers in co-transfection assays (Figure 13C).^85^ When treated with LG-100754, db/db animals had an improvement in insulin resistance in vivo. Therefore, the ability of LG-100754 to increase PPAR**γ** sensitivity and relieve insulin resistance proves that it can be an effective agent in the management of type 3 diabetes mellitus.

### nTZDpa

Chemically, nTZDpa is 5-chloro-1-(4-chlorobenzyl)-3-(phenylthio)-1H-indole-2-carboxylic acid. This compound is selectively non-thiazolidinedione PPAR**γ** partial agonist (Figure 13D) and produces 25% of maximum efficacy. It acts differently from full PPAR**γ** agonists by producing distorted receptor conformation, amendable adipocyte development, and gene expression. It also modulates metabolism and insulin sensitivity without causing cardiac hypertrophy in mice using in vivo studies.^86^ Chronic treatment of nTZDpa or a TZD full agonist on fat-fed C57BL/6J mice showed a reduction in hyperglycemia and hyperinsulinemia.

### SR-1664

Chemically, SR-1664 is (S)-4’-((2,3-dimethyl-5-((1-(4nitrophenyl)ethyl)carbamoyl)-1H-indol-1-yl)methyl)-[1,1’-biphenyl]-2-carboxylic acid. It is a potential antidiabetic agent that binds to PPARγ and inhibits phosphorylation of Cdk5-mediated PPARγ (IC_50_ = 80 nM; K_i_ = 28.67 nM) without exhibiting PPARγ agonist activity (Figure 13E). The agent is sought to reduce fasting insulin levels and shows improvement in insulin sensitivity in a diabetic mouse model.^87^ This compound causes fluid retention and weight gain with potent antidiabetic activity.

### WY-14643

It is a selective PPARα agonist. Chemically, it is 2-((4-chloro-6-((2,3-dimethylphenyl)amino)pyrimidin-2-yl)thio)acetic acid (Figure 13F). It is shown to increase in vitro insulin signaling and uptake of glucose in the skeletal muscle cells by the action of insulin.^88^ It has also been observed that WY-14643 tends to ameliorate the memory impairments in mice induced by scopolamine by the activation of PPARα and mediation of brain-derived neurotrophic factor pathway.^89^ Further, other reported studies such as attenuation of neurodegeneration caused by amyloid β in vitro and prevention of neuroinflammation also points toward the high probability of its effectiveness in type 3 diabetes mellitus.^90,91^

## Dipeptidyl Peptidase IV (DPP-IV) Inhibitors

Dipeptidyl peptidase IV (DPP-IV) is the category of DPP-IV activity and structure homologue proteins that is responsible for breakdown of different proline dipeptidase via serine dipeptidyl peptidase proline enzyme includes fibroblast activation protein and attractin. DPP-IV inhibitors is mediated via stabilizing of the incretin hormones glucagon-like peptidase-I and glucose-dependent insulinotropic polypeptide resulting in glucose-dependent insulin secretion.^92^

### DPPI 1c Hydrochloride

**Figure 14.**
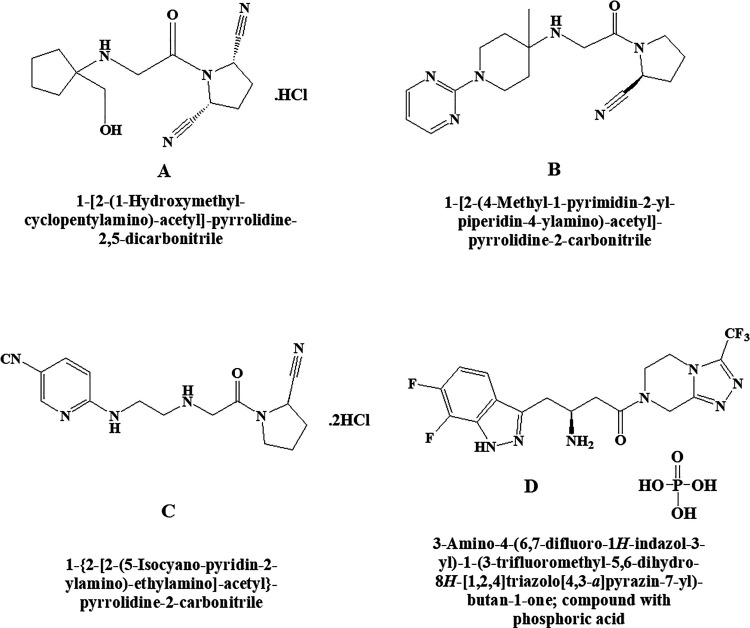
Dipeptidyl peptidase IV inhibitors (A) DPPI 1c hydrochloride, (B) K-579, (C) NVP DPP 728 dihydrochloride, and (D) PK 44 phosphate.

Chemically, DPPI 1c hydrochloride is (2S,5R)-1-(2-((1-(hydroxymethyl)cyclopentyl)amino)acetyl)pyrrolidine-2,5-dicarbonitrile hydrochloride (Figure 14A). It was found that the glucagon-like peptide-1 (GLP-1) inhibitors that degrade the DPP-IV enzyme are effective in treating type 2 diabetes in preclinical and clinical models. It further demonstrated that DPPI1c acts as a GLP-1 inhibitor by inhibiting DPP-IV enzyme which is further helpful for the treatment of type 2 diabetes mellitus.^93^ Increase in plasma GLP-1 levels and improved glucose tolerance in diabetic mice was reported after the treatment with DPPI 1c hydrochloride.

### K-579

This is an inhibitor of dipeptidyl peptidase IV and having a slow-binding capacity. Chemically, it is (S)-1-(2-((4-methyl-1-(pyrimidin-2-yl)piperidin-4-yl)amino)acetyl)pyrrolidine-2-carbonitrile. It is a potent long-acting hypoglycemic agent reported by Takasaki et al (Figure 14B).^94^ Prior administration of K-579 to Zucker fatty rats attenuated the glucose expedition after the first and second glucose loading without inducing hypoglycemia. After 8 hours of administration, K-579 inhibited the plasma DPP-IV activity and significantly decreased blood glucose level in glibenclamide pretreated rats.

### NVP DPP 728 Dihydrochloride

Chemically, NVP DPP 728 dihydrochloride is (R)-6-((2-((2-(2-cyanopyrrolidin-1-yl)-2-oxoethyl)amino)ethyl)amino)nicotinonitrile. This drug also belongs to DPP-IV and acts as an inhibitor having 15 000-fold more than DPP-II (Figure 14C). In vivo studies demonstrated the hypoglycemic like effect improves glucose tolerance, increases insulin, GLUT2, and GLP-1 levels augment insulin secretion and reduce (preserves) islet size.^95^ Effects of aging from glucose metabolism after the oral glucose challenge test were reported in aged DPP-IV positive (+) Fischer 344 (F344) and DPP-IV deficient (−) F344. These results indicated that treatment with NVP-DPP728 ameliorates glucose tolerance via direct inhibition of plasma DPP-IV activity in specific DPP-IV (+) F344 aged rats.^95^

### PK 44 Phosphate

PK 44 phosphate is an indole and indazole-based structure belonging to DPP-IV inhibitor type of effect (IC_50_ = 15.8 nM) reported by Tozer^96^ in the treatment of type 2 diabetes mellitus. Chemically, PK 44 phosphate is (S)-3-amino-4-(6,7-difluoro-1H-indazol-3-yl)-1-(3-(trifluoromethyl)-5,6-dihydro-[1,2,4]triazolo[4,3-a]pyrazin-7(8H)-yl)butan-1-one phosphate (Figure 14D). It also showed 1000-fold selectivity for DPP-IV rather than DPP-8 and DPP-9, which improves glucose tolerance in a mouse model.

### Other Compounds

#### AS-1949490

**Figure 15.**
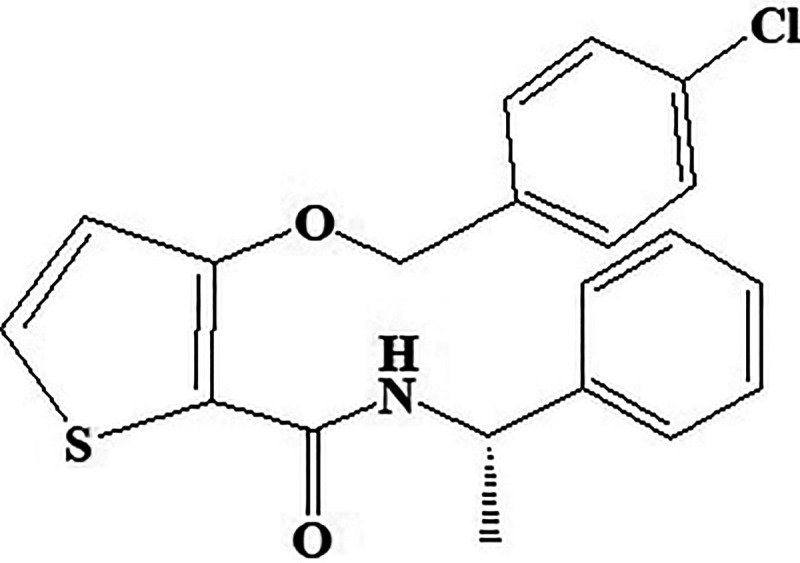
Inositol 5’-phosphatase 2 inhibitor AS-1949490.

Potent novel molecule AS-1949490 acts as SH2 domain-containing inositol 5’-phosphatase 2 inhibitor showed significant selectivity over other related phosphatases (Figure 15) and has 30 times more affinity for SH2 domain-containing inositol 5’-phosphatase 2 (SHIP2) than SHIP1. Chemically, AS-1949490 is (S)-3-((4-chlorobenzyl)oxy)-N-(1-phenylethyl)thiophene-2-carboxamide. In vitro and in vivo data exhibited antidiabetic effects in L6 myotubules by stimulating glucose metabolism increases insulin-induced phosphorylation and regulating gluconeogenesis. Scientist^97^ reported lipid SHIP2 regulates insulin signals. Therefore, it can be concluded from the studies that pharmacological inhibition of SHIP2 activates glucose metabolism through gene expression.

#### Donepezil

**Figure 16.**
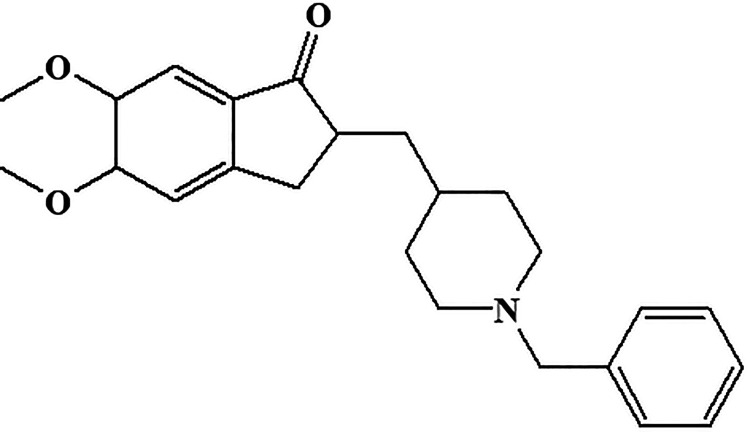
Central acetylcholinesterase inhibitor donepezil.

Chemically, donepezil is 2-((1-benzylpiperidin-4-yl)methyl)-5,6-dimethoxy-2,3-dihydro-1H-inden-1-one (Figure 16). It belongs to reversible acetylcholinesterase inhibitor and acts centrally whose main therapeutic use is in the treatment of Alzheimer disease. In early 2006, the donepezil was reported for improving cognitive function in the patients who had Alzheimer types of symptoms.^98^ Therefore, many of the physicians, psychiatrists, and neurologists used to prescribe donepezil in patients with Alzheimer disease.

#### Amlexanox

**Figure 17.**
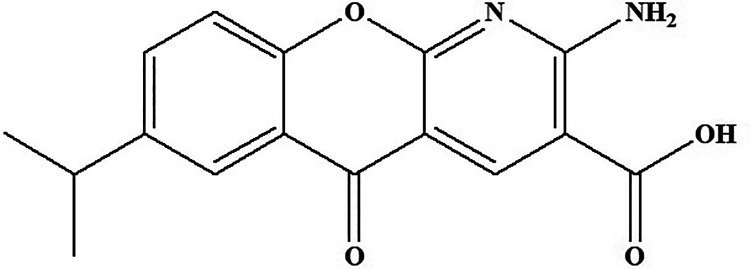
TANK-binding kinase 1 compound amlexanox.

Amlexanox belongs to the class of inhibitors of TANK-binding kinase 1 (TBK1) and IKKε (Figure 16) reported by Reilly (2012).^99^ Chemically, it is 2-amino-7-isopropyl-5-oxo-5H-chromeno[2,3-b]pyridine-3-carboxylic acid (Figure 17). There are numerous evidences that support a link between obesity and insulin resistance through inflammation processes. Through high-fat diet feeding, both the targeting sites are induced in fat and liver by NF-κB activation, which in turn initiates counter inflammation that preserves energy. Treatment with amlexanox accelerates the energy expenditure through increased thermogenesis, producing weight loss, improved insulin sensitivity, and decreased steatosis in obese mice.^100^ In vitro studies reported the inhibition of c-terminal chaperone activity via binding of Hsp90 with amlexanox.

## Conclusion

Type 3 diabetes (Alzheimer associated with diabetes mellitus) is fast aging disease now days. It is one of the major prevalent diseases that affect most of the diabetic individuals. Scientist of R&D of every pharmaceutical or biotechnology industry is searching new molecules that can treat this disease. This review article may give a new light into the medical field that can start for the clinical trials or post market surveillance, so that among these, a new molecule may be established as a neuroprotective agents in the market. Future scientists who want to be aware of disease of diabetes and diabetes-induced Alzheimer may analyze the potency of these molecules for the treatment though different softwares like molecular docking or Prediction of activity spectra for substances (PASS). The literature signifies that there are many new novel molecules developed by research centers that are still unfolded and required to be tested for preclinical and clinical trials.

## Supplemental Material

Supplemental Material, Graphical_Abstract - Unwinding Complexities of Diabetic Alzheimer by Potent Novel Molecules Click here for additional data file. Supplemental Material, Graphical_Abstract for Unwinding Complexities of Diabetic Alzheimer by Potent Novel Molecules by Sumeet Gupta, Anroop Nair, Vikas Jhawat, Nazia Mustaq, Abhishek Sharma, Meenakshi Dhanawat and Shah Alam Khan in American Journal of Alzheimer's Disease & Other Dementias
