# Supplementary figures and images for: Unwinding Complexities of Diabetic Alzheimer by Potent Novel Molecules
Source: Am J Alzheimers Dis Other Demen. 2020 Aug 31;35:1533317520937542. doi: 10.1177/1533317520937542 (PMC10623924; doi:10.1177/1533317520937542)

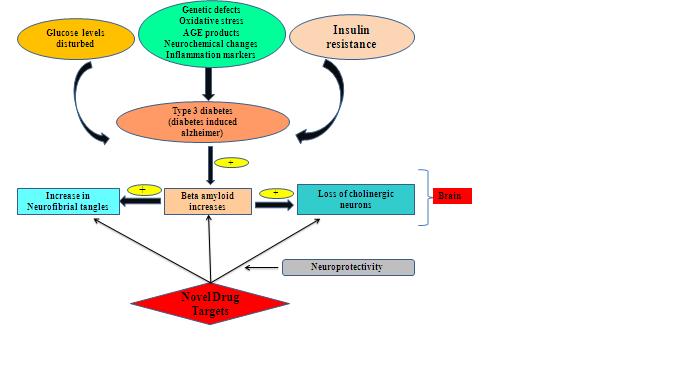

Supplement: Supplemental Material, Graphical_Abstract - Unwinding Complexities of Diabetic Alzheimer by Potent Novel Molecules [file Graphical_Abstract.jpg]
